# CREATING A DOCUMENTARY CAPTURING THE NARRATIVES OF MEN ATTENDING IRISH MEN’S SHEDS

**DOI:** 10.1093/geroni/igad104.0787

**Published:** 2023-12-21

**Authors:** Melinda Heinz, Laura Gleissner

**Affiliations:** Upper Iowa University, Fayette, Iowa, United States; University of Northern Iowa, Cedar Falls, Iowa, United States

## Abstract

How do we engage more individuals to consider and care about issues impacting older adults? Diversifying the mediums of how older adult stories are told offers one solution. Filmmaking is one medium where the narratives of older adults can be captured. Seeing and hearing directly from older adults speak about the issues that affect them provides a distinct way to understand experiences of aging. I interviewed men in County Limerick about how Men’s Sheds promoted purpose and meaning in the lives of older men. After transcribing each interview, I partnered with a filmmaker to help create a documentary to tell the story of how Men’s Sheds create an inclusive atmosphere for older adult men to continue feeling useful in older adulthood. This presentation will explain how researchers who conduct interviews with older adults are uniquely positioned to collaborate with filmmakers to help tell the stories of older adults.

